# Pharmacists’ perceived knowledge, attitudes and practice in geriatrics: findings from a multinational multilingual open online survey

**DOI:** 10.1007/s41999-026-01504-z

**Published:** 2026-05-14

**Authors:** Klejda Harasani, Giulia Ogliari, Luca Soraci, Karolina Piotrowicz, Sofia Duque, Mitilda Gugu, Katarina Stefanović, Marina Kotsani

**Affiliations:** 1 Department of Pharmacy, Faculty of Medicine, University of Medicine, Tirana, Tirana, Albania; 2 National Institute for Health and Care Research (NIHR) Health Determinants Research Collaboration (HDRC), Leicestershire County Council, Glenfield, Leicestershire UK; 3 Unit of Geriatric Medicine, Italian National Research Center on Aging (IRCCS INRCA), Cosenza, Italy; 4 Department of Internal Medicine and Gerontology, Faculty of Medicine, Jagiellonian University Medical College, Kraków, Poland; 5 Preventive Medicine and Public Health Institute, Faculty of Medicine, University of Lisbon, Lisbon, Portugal; 6 Preclinical Department, Faculty of Technical Medical Sciences, University of Elbasan “Aleksandër Xhuvani”, Elbasan, Albania; 7 Geriatric Department, Clinical Hospital Zvezdara, Belgrade, Serbia; 8 Hellenic Society for the Study and Research of Ageing, Athens, Greece

**Keywords:** Pharmacist, Geriatric medicine, Educational training, Europe

## Abstract

**Purpose:**

This study aims to assess pharmacists´ self-perceived knowledge or competence of major geriatric topics and skills, the perceived relevance of these to work or clinical practice, and their interest in receiving further education or training.

**Methods:**

A survey was developed in English through a co-creative process that involved clinicians, researchers of the COST Action 21122 PROGRAMMING and international stakeholders from multiple professional background. It was translated into 24 languages and disseminated online. Pharmacists’ perceived knowledge, relevance, and interest in 33 geriatric topics were assessed using 5-point Likert scales. The median and modal response for each topic, as well as a global topic score (relevance + interest − knowledge) were calculated. Composite scores were standardized by computing *Z*-scores as (global topic score − mean global topic score across the 33 topics) divided by the standard deviation of global topic scores. Topics were then ranked and categorized into quintiles.

**Results:**

A total of 346 pharmacists across Europe, predominantly women (82.4%), completed the survey. Mean relevance and interest scores were consistently higher than self-reported knowledge across the majority of topics. Polypharmacy and deprescribing (*Z* score: 2.45)*,* chronic pain (*Z* score: 1.31), and depression were the top-ranked training priorities. Assessing older people in emergency departments and gerodontology were the lowest ranked.

**Conclusion:**

Among pharmacists participating across Europe, self-perceived knowledge in geriatrics was consistently lower than the perceived relevance and interest across most topics, indicating important perceived educational gaps. These findings support the need to strengthen geriatric competencies within pharmacy education and professional development.

**Supplementary Information:**

The online version contains supplementary material available at https://doi.org/10.1007/s41999-026-01504-z.

## Key summary points

**Aim:**

To assess pharmacists´ self-perceived knowledge or competence of major geriatric topics and skills, the perceived relevance of these to work or clinical practice, and their interest in receiving further education or training.

**Findings:**

A total of 346 pharmacists mainly from European countries completed the online survey of the COST Action PROGRAMMING CA21122. They reported modest self-rated knowledge across all topics, while both perceived relevance and interest were consistently higher.

**Message:**

In this online survey of pharmacists-based primarily in European countries, polypharmacy and deprescribing, chronic pain, and depression were the top-ranked training priorities.

**Supplementary Information:**

The online version contains supplementary material available at https://doi.org/10.1007/s41999-026-01504-z.

## Introduction

Pharmacists provide care to older people across nearly all healthcare settings, including hospitals, primary care, community pharmacies, long-term care facilities, and other outpatient clinics, where they contribute to patients’ education and medication management with pharmacotherapy optimization. Almost all (96.6%) middle-aged and older adults visit a pharmacy annually, with nearly one fifth utilizing at least one specified pharmacy service [1]. A recent review revealed that the community pharmacy represents the primary source of drugs for self-medication among the older population [2]. According to European Commission statistics, in 2023, over 418,000 pharmacists were practicing across 26 EU countries, underscoring the substantial size of the pharmacy workforce and its potential impact on the care of ageing populations [3].

More than half of the older population in four of six countries are prescribed five or more medications, highlighting the growing complexity of pharmacotherapy and the importance of pharmacists as an integral part of interprofessional teams in providing expertise in medication reconciliation, prescription optimization, management of polypharmacy, follow-up and monitoring [4, 5]. Pharmacists´ interventions on geriatric patients in hospital wards across Europe have led to improved appropriateness of treatments, increased drug safety and reduced medication-related admissions [6]. Potentially inappropriate prescribing (PIP) is the most significant issue in this age group [7]. Although geriatric pharmacy has been formally recognized as a specialty for several decades, its integration into pharmacy education and practice remains inconsistent across countries. Allied healthcare professionals—including pharmacists—often lack sufficient depth of knowledge and practical skills in geriatric medicine to deliver optimal care to older adults across all settings [8–10]. This need for stronger geriatric competences among all healthcare professionals, not only geriatricians, is particularly pressing in countries where geriatric medicine is still emerging and not formally recognized as a specialty. Furthermore, the inclusion of geriatric courses in pharmacy curricula worldwide remains limited, leaving future pharmacists insufficiently prepared to meet the complex needs of an increasingly ageing population [8–12]. To date, no multi-country or large-scale international studies have systematically examined pharmacists’ knowledge, attitudes, and practices in geriatrics, nor their educational needs in geriatric medicine, particularly across Europe. Despite pharmacists’ recognized involvement in the care of older adults across Europe [13, 14] there is limited empirical evidence regarding their self-perceived geriatric knowledge, competencies, and attitudes [15].

Therefore, we aimed to assess pharmacists´ self-perceived knowledge and competencies of major geriatric topics and skills, the perceived relevance of these to clinical practice, and the interest in further education or training on them, by exploring the responses of pharmacists who completed the open online survey on educational needs led by the COST Action 21122 PROmoting GeRiAtric Medicine in countries where it is still eMergING (PROGRAMMING) [16, 17]. In addition, we explored the pharmacists´ attitudes towards caring for older adults, their interest in geriatric training, perceived barriers to attending such courses, and whether these differed by age and gender.

## Methods

### Development, content and dissemination of the survey

The open online survey was developed through an iterative co-creative process that involved clinicians, researchers of the COST Action 21122 PROGRAMMING as well as international stakeholders from multiple professional backgrounds. The structure and content of the survey were discussed by PROGRAMMING members with an external panel of 68 international stakeholders from 24 countries and then revised based on their feedback. This co-creation process took place from October 2022 to May 2023 [16].

The survey targeted professionals and medical students. In detail, healthcare professionals completed these sections: Informed Consent, Demographics, Topics and Skills, Current Profession, Previous Education in Geriatric Medicine, Interest in Care of Older People or Geriatric Medicine, Suggestions for Courses in Care for Older People or Geriatric Medicine, and Closure.

The section Topics and Skills listed 33 key geriatric topics and skills (full list available in [16]). These topics and skills included key domains of geriatric care, including concepts of ageing, common geriatric syndromes, clinical assessment and management skills, as well as communication competencies. For each topic or skill, the professionals rated their knowledge or competence on a 5-point Likert scale ranging from very low (1) to very high (5). Similarly, on 5-point Likert scales, they rated the relevance of this topic or skill to their current or future work or clinical practice and their interest in further training on each topic or skill, respectively.

The survey was developed in English and later translated into 24 other languages. It was disseminated online between October 9, 2023, and June 5, 2024, via multiple strategies: official website, emails to national and international stakeholders, social media, and professional contacts. A more detailed description of the mixed-method survey protocol can be found in the published article by Ogliari et al. [16].

Ethics approval was obtained in the countries where local regulations required for this, while local regulations and institutional policies were followed in the other countries as previously detailed (Ogliari et al., 2025).

### Data analysis

Descriptive statistics were used to summarize the characteristics of participating pharmacists. Continuous variables were described with mean and standard deviation (SD), while categorical variables were reported as absolute frequencies and percentages. For subgroup analyses, age was categorized into tertiles (23–30 years, 31–42 years, and 43–72 years)**.** Associations between categorical variables (e.g., exposure to geriatric medicine [GM] courses, clinical rotations, barriers to education) and sociodemographic characteristics (age tertiles, sex) were assessed using Pearson’s chi-square test across all categories of the variables. Fisher’s exact test was used when expected cell counts were small.

For each of the 33 geriatric topics and skills, we calculated pharmacists’ perceived mean knowledge score and standard deviation (SD), perceived mean (SD) relevance score and perceived mean (SD) interest score, respectively.

In addition to reporting mean values with standard deviations, we also summarized the distribution of responses using the median and the modal response (i.e., the most frequently selected Likert category) for each topic, to better capture both the central tendency and the most common perception among respondents. To quantify training priorities across different geriatric medicine topics, we developed a global topic score integrating the three dimensions assessed in the survey using the formula:

$${\mathrm{global}}\;{\text{topic score}}\, = \,{\text{perceived relevance score}}\, + \,{\text{perceived interest score}}{-}{\text{perceived knowledge score}}.$$

This approach emphasizes areas where perceived importance and interest exceed current knowledge, identifying potential training needs; more specifically, topics characterized by higher global scores were those with a higher gap between interest/relevance and knowledge.

For standardization and comparability of educational needs across topics, we computed standardized *Z*-scores of the global topic score. We computed a *Z* score for each global topic score. For example, the *Z* score for CGA was computed as:

$${\text{Z score for CGA}}\, = \,\left[ {\left( {\text{CGA global topic score}} \right) - \left( {\text{the mean of all 33 global topic scores}} \right)} \right]/({\text{SD of all 33 global topic scores}}).$$

*Z*-scores were calculated relative to the study sample’s mean and SD of all 33 global topic scores and are therefore sample-dependent.

Topics were then ranked according to their *Z*-scores and categorized into quintiles. All analyses were performed using R version 4.6, with a two-sided *p* value < 0.05 considered statistically significant.

## Results

### Sociodemographic characteristics

The survey received 6,099 responses; after cleaning, there were 5922 responses including 5474 from professionals and 448 from medical students. Of the 5474 from professionals, 346 (6.3%) were from pharmacists and represented the analytical sample of this study.

They were predominantly women (285; 82.4%) and the mean age was 38.8 years (SD 11.2).

Among them, 339 had the main degree in pharmacy, while the other 7 reported another main qualification or degree in another setting (*n* = 1 in dentistry, *n* = 1 in medicine, *n* = 1 in podiatry, *n* = 1 in speech and language therapy, *n* = 3 in other degrees). The main characteristics of the study population are reported in Table 1.

**Table 1:** General characteristics of pharmacists participating in the survey

	*N* = 346
Age, years mean (SD)	38.8 (11.2)
Gender, *n* (%)	
Men	60 (17.3)
Women	285 (82.4)
Prefer not to say	1 (0.3)
Country of study for the main degree, *n* (%)	
Albania	96 (27.7)
Türkiye	83 (24.0)
Croatia	61 (17.6)
Serbia	46 (13.3)
Italy	9 (2.6)
Portugal	9 (2.6)
Poland	7 (2.0)
United Kingdom	6 (1.7)
Greece	5 (1.4)
Bosnia and Herzegovina	4 (1.2)
Belgium	3 (0.9)
Countries with less than 3 pharmacists are listed in the footnote	17 (4.9)
Country of work at present, *n* (%)	
Albania	83 (24.0)
Türkiye	83 (24.0)
Croatia	68 (19.7)
Serbia	47 (13.6)
Portugal	9 (2.6)
Poland	8 (2.3)
Germany	6 (1.7)
United Kingdom	5 (1.4)
Greece	4 (1.2)
Italy	4 (1.2)
France	3 (0.9)
Kosovo^a^	3 (0.9)
Countries with less than 3 pharmacists are listed in the footnote	23 (6.6)
Professionals with international mobility, *n* (%)	39 (11.3)
Mobility pattern, *n* (%)	
Albania, overall	16 (41)
Albania—Germany	4 (10.3)
Albania—Italy	3 (7.7)
Albania—Kosovo^a^	2 (5.1)
Albania—other countries with less than 2 mobilities	7 (17.9)
Italy, overall	8 (20.5)
Italy—Croatia	5 (12.8)
Italy—Albania	2 (5.1)
Italy—Poland	1 (2.6)
Working in a primary care setting, *n* (%)	235 (67.9)
Working in a secondary care setting, *n* (%)	112 (32.4)
University/academia	9 (2.6)
Pharmaceutical industry, *n* (%)	4 (1.2)
More than 1 setting, *n* (%)	15 (4.3)

Among the reported countries of study for the main degree/qualification, Germany, Spain, Sweden and other countries/more than 1 country were each indicated by two respondents, while Bulgaria, France, Kosovo*, Montenegro, the Netherlands, North Macedonia, Romania, Slovakia, and Slovenia were each indicated by one respondent. Among the reported countries of current work, Austria, Belgium, Cyprus, Czech Republic, the Netherlands, Spain, Sweden, and the United States of America had 2 professionals; Bosnia and Herzegovina, Bulgaria, Denmark, Kuwait, Montenegro, North Macedonia, Romania had 1 professional

Primary care settings refer to community pharmacy or other primary care or community settings. Secondary care settings refer to outpatient specialist clinics, acute general hospital, acute psychiatric hospital and other secondary care settings

*SD* standard deviation, *n* number

^a^This designation is without prejudice to positions on status, and is in line with UNSCR 1244/1999 and the ICJ Opinion on the Kosovo declaration of independence

They reported studying for the main degree in 23 countries including Albania (27.7%), Türkiye (24.0%), Croatia (17.6%), Serbia (13.3%), Italy (2.6%), Portugal (2.6%), Poland (2.0%), UK (1.7%), and other countries (Table 1). They reported currently working in 27 different countries or regions. The four countries with the highest number of pharmacists working there are Albania (24.0%), Türkiye (24.0%), Croatia (19.7%) and Serbia (13.6%). Of note, 39 (11.3%) pharmacists worked in a different country from the one where they studied for their main degree; the main mobility patterns started from Albania towards Germany, Italy, Kosovo*, other countries, followed by Italy towards Croatia, Albania, and Poland.

The majority of the participants worked in primary care or community setting (*n* = 235, 67.9%), mostly declaring to work at local pharmacies (*n* = 217); other 112 (32.4%) participants declared to work in acute or secondary care settings with 75 declaring to work in pharmacies; the remaining declared to work in other settings (mainly university/academia and pharmaceutical companies). Finally, 15 participants (4.3%) declared to work in more than one setting.

### Previous exposure to geriatric medicine

Among the 346 pharmacists, previous education in geriatric medicine (GM) during formal professional or undergraduate university education was generally limited (Table 2). Overall, only one‐third of the respondents reported having attended formal GM courses (32.4%), while the majority had either never attended such a course (60.4%) or were unsure (7.2%). Practical exposure through clinical rotations in geriatric settings was even more infrequent. Less than 10% of the study population had completed a rotation in an acute geriatric hospital ward, and only a very small minority had experience in rehabilitation settings (2.0%), care or nursing homes (4.3%) or outpatient geriatric clinics (6.4%). A similar pattern was observed for extracurricular activities, with approximately one in five reporting volunteer work or research work related to geriatric medicine.

**Table 2:** Previous attendance of courses in GM in the whole study population and across age tertiles

	Age tertiles				
	All (*n* = 346)	Youngest 23–30 years (*n* = 130)	Middle 31–42 years (*n* = 101)	Oldest 43–72 years (*n* = 115)	*p* value
Attended courses in GM					0.224
Yes	112 (32.4)	38 (29.2)	33 (32.7)	41 (35.6)	
No	209 (60.4)	87 (66.9)	59 (58.4)	63 (54.8)	
Not sure	25 (7.2)	5 (3.8)	9 (8.9)	11 (9.6)	
Rotations in GM acute hospital ward					0.726
Yes	28 (8.1)	12 (9.2)	10 (9.9)	6 (5.2)	
No	299 (86.4)	111 (85.4)	85 (84.2)	103 (89.6)	
Not sure	19 (5.5)	7 (5.4)	6 (5.9)	6 (5.2)	
Rotations in rehabilitation setting, *n* (%)					0.970
Yes	7 (2.0)	2 (1.5)	2 (2.0)	3 (2.6)	
No	321 (92.8)	122 (93.9)	93 (92.1)	106 (92.2)	
Not sure	18 (5.2)	6 (4.6)	6 (6.0)	6 (5.2)	
Rotations in care home or nursing home, *n* (%)					0.637
Yes	15 (4.3)	5 (3.8)	7 (6.9)	3 (2.6)	
No	314 (90.8)	118 (90.8)	89 (88.1)	107 (93.1)	
Not sure	17 (4.9)	7 (5.4)	5 (5.0)	5 (4.3)	
Rotations in outpatient clinic, *n* (%)					0.777
Yes	22 (6.4)	11 (8.5)	6 (5.9)	5 (4.3)	
No	308 (89.0)	113 (86.9)	90 (89.1)	105 (91.4)	
Not sure	16 (4.6)	6 (4.6)	5 (4.9)	5 (4.3)	
Volunteer work, *n* (%)					0.374
Yes	76 (22.0)	36 (27.7)	19 (18.8)	21 (18.3)	
No	258 (74.6)	90 (69.2)	79 (78.2)	89 (77.4)	
Not sure	12 (3.4)	4 (3.1)	3 (3.0)	5 (4.3)	
Research work, *n* (%)					0.855
Yes	79 (22.8)	30 (23.1)	20 (19.8)	29 (25.2)	
No	254 (73.4)	94 (72.3)	78 (77.2)	82 (71.3)	
Not sure	13 (3.8)	6 (4.6)	3 (3.0)	4 (3.5)	

*p* value derived from Chi-squared test or exact Fisher’s test when appropriate (for within group small counts)

Stratification by age tertiles has also been reported (Table 2). Albeit not reaching statistical significance, participants in the youngest age group (23–30 years) were less likely to have attended a GM course compared with their older counterparts. However, this younger group showed marginally higher exposure to clinical rotations in all GM settings, particularly in acute geriatric wards (9.2%) and outpatient clinics (8.5%). By contrast, respondents in the oldest tertile (43–72 years), despite reporting the highest attendance of GM courses (35.6%), had the lowest prevalence of practical GM rotations, with only 5.2% having rotated in an acute ward and 4.3% in an outpatient clinic. Volunteer and research activities followed a similar age‐related trend: engagement in volunteer work was more common among younger participants (27.7% in the 23–30 year tertile) and progressively decreased with age, whereas participation in research was slightly higher in the oldest group (25.2%) compared with those aged 23–30 (23.1%) and 31–42 (19.8%).

Stratification by sex groups is reported in Supplementary Table 1 and showed a significantly lower prevalence of GM rotations in acute care wards among women compared to men (6.7% vs 15.0%, respectively; *p* = 0.047).

### Interest in care of older people

Interest in care of older people and in the whole population and by age tertiles is reported in Supplementary Table 2.

In the overall study population, the majority of participants reported positive attitudes toward older people and expressed substantial interest in geriatric medicine education. More specifically, 41.3% of respondents stated that they *highly or very highly* enjoyed engaging with older adults, in both professional and non-professional settings. Nevertheless, the perceived social prestige associated with caring for older people was low, with 84.1% rating it as *very little* or *fairly* prestigious. When stratified by age tertiles, only minor and nonsignificant differences were observed in general attitudes. The proportion of participants who highly enjoyed engaging older adults ranged from 38.5% in the youngest age group (23–30 years) to 47.0% in the oldest group (43–72 years).

### Interest in courses in GM

Overall and age-stratified interest in courses in GM is reported in Table 3.

**Table 3:** Interest in courses in GM in the whole study population and across age tertiles

	All (*n* = 346)	23–30 years (*n* = 130)	31–42 years (*n* = 101)	43–72 years (*n* = 115)	*p* value
Interest in attending courses in GM, *n* (%)					0.305
Very little-fairly	166 (48.0)	69 (53.1)	47 (46.5)	50 (43.5)	
Much-very much	180 (52.0)	61 (46.9)	54 (53.5)	65 (56.5)	
Preferred type of course, *n* (%)					**0.022**
In-person	44 (12.7)	10 (7.7)	10 (9.9)	24 (20.9)	
Online	131 (37.9)	51 (39.2)	43 (42.6)	37 (32.2)	
Hybrid	171 (49.4)	69 (53.1)	48 (47.5)	54 (46.9)	
*Barriers, n (%)*					
Financial loss					**0.042**
Yes	139 (40.2)	65 (50.0)	39 (38.6)	35 (30.4)	
No	138 (39.9)	44 (33.8)	41 (40.6)	53 (46.1)	
Not sure	69 (19.9)	21 (16.2)	21 (20.8)	27 (23.5)	
Paid leave issues					** < 0.001**
Yes	143 (41.3)	69 (53.1)	44 (43.6)	30 (26.1)	
No	140 (40.5)	42 (32.3)	41 (40.6)	57 (49.6)	
Not sure	63 (18.2)	19 (14.6)	16 (15.8)	28 (24.3)	
Doubts about feasibility of taught skills					**0.004**
Yes	97 (28.0)	42 (32.3)	25 (24.7)	30 (26.1)	
No	177 (51.2)	61 (46.9)	65 (64.4)	51 (44.3)	
Not sure	72 (20.8)	27 (20.8)	11 (10.9)	34 (29.6)	
Doubts about the relevance of taught skills					0.056
Yes	68 (19.7)	24 (18.5)	18 (17.8)	26 (22.6)	
No	223 (64.4)	87 (66.9)	73 (72.3)	63 (54.8)	
Not sure	55 (15.9)	19 (14.6)	10 (9.9)	26 (22.6)	
Doubts about the necessity to improve skills					**0.005**
Yes	47 (13.6)	20 (15.4)	14 (13.9)	13 (11.4)	
No	250 (72.2)	98 (75.4)	78 (77.2)	74 (64.3)	
Not sure	49 (14.2)	12 (9.2)	9 (8.9)	28 (24.3)	
Language barrier					** < 0.001**
Yes	65 (18.8)	11 (8.5)	16 (15.8)	38 (33.0)	
No	237 (68.5)	99 (76.1)	77 (76.2)	61 (53.0)	
Not sure	44 (12.7)	20 (15.4)	8 (7.9)	16 (13.9)	
Low motivation					0.338
Yes	40 (11.6)	12 (9.2)	14 (13.9)	14 (12.2)	
No	260 (75.1)	101 (77.7)	78 (77.2)	81 (70.4)	
Not sure	46 (13.3)	17 (13.1)	9 (8.9)	20 (17.4)	
Time constraints					**0.007**
Yes	218 (63.0)	91 (70.0)	61 (60.4)	66 (57.4)	
No	85 (24.6)	23 (17.7)	34 (33.7)	28 (24.3)	
Not sure	43 (12.4)	16 (12.3)	6 (5.9)	21 (18.3)	

In bold, p values considered statistically significant

More than half of participants were *highly* interested in attending courses in geriatric medicine. With respect to educational preferences, most respondents were in favor of hybrid or online course formats, whereas in-person-only courses were selected by a small proportion. Several barriers to training were highlighted. The most commonly reported obstacles were time constraints (63.0%), paid leave issues (41.3%), and financial loss (40.2%). Doubts about the feasibility and the relevance of the taught skills were reported by 28.0% and 19.7% of participants, respectively. A language barrier in case the course is in English was perceived by 18.8% of respondents, whereas the proportion reporting low motivation to attend courses was relatively small (11.6%). Stratification by age tertiles showed that the interest in attending GM courses was slightly lower in younger respondents (46.9%) and progressively increased with age (56.5% in the oldest group). Preference for training format varied by age. Older participants more frequently selected in-person courses, whereas younger respondents showed a stronger preference for hybrid format (*p* = 0.022).

Importantly, several barriers differed substantially across age tertiles. Younger respondents were more likely to report financial loss (50.0%), paid leave issues (53.1%), and time constraints (70.0%) as limiting factors for attending GM courses, while language barriers were more commonly reported in the oldest age group (33.0% vs 8.5% in the youngest age group, *p* < 0.001). Differences were also observed in the feasibility of the taught skills with younger participants expressing greater concern, whereas doubts about the necessity to improve skills were particularly frequent in the oldest group (*p* = 0.005).

Stratification by sex groups (Supplementary Table 3) did not show any significant differences between men and women.

Stratification by sex groups (Supplementary Table 4) did not show any significant differences between men and women.

### Distribution of pharmacists’ responses

**Fig. 1:**
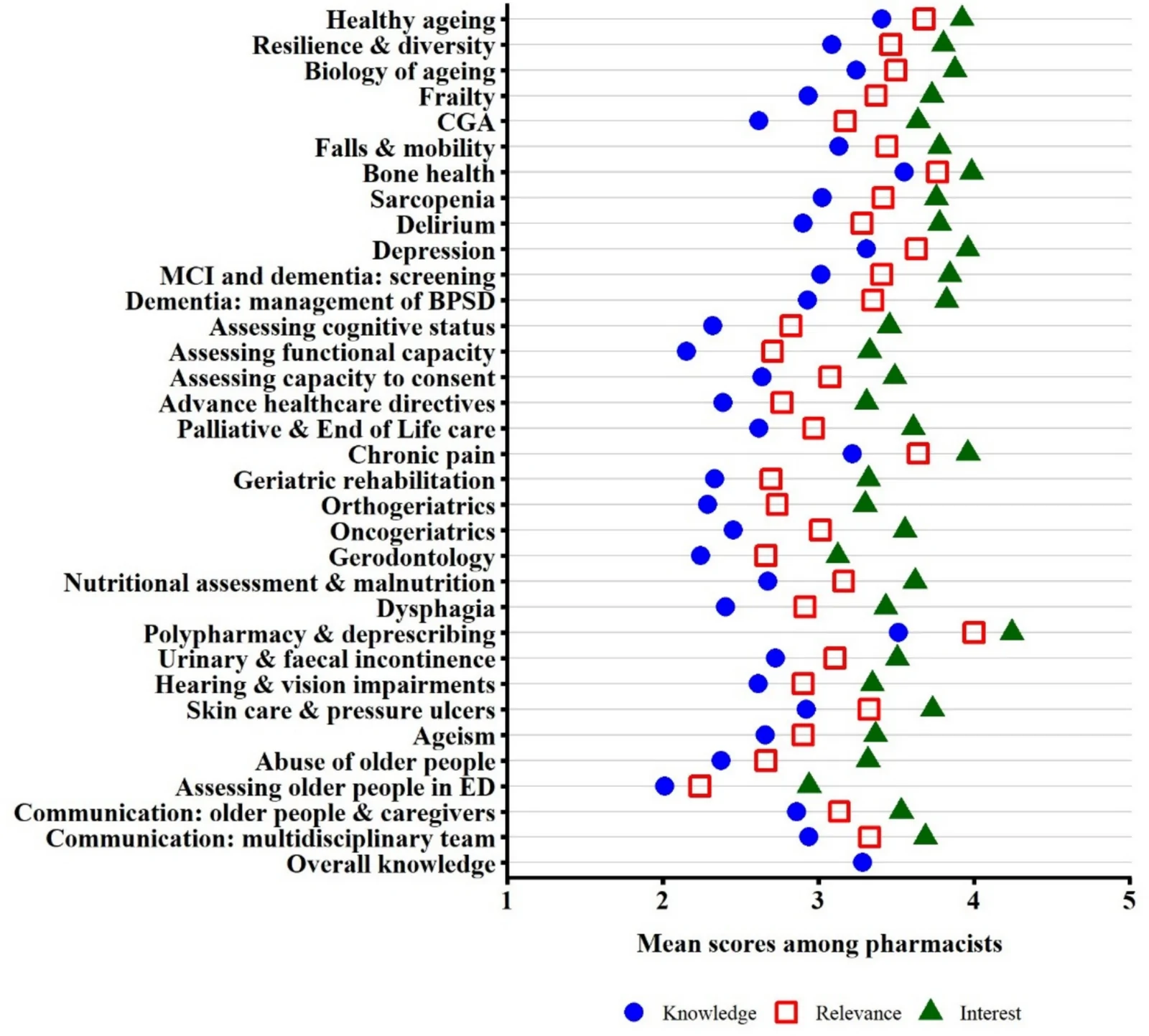
Pharmacists’ perceived knowledge, relevance and interest by topics or skill. *ED* emergency department, *CGA* comprehensive geriatric assessment, *BPSD* behavioral and psychological symptoms of dementia, *MCI* mild cognitive impairment

Figure 1 shows the mean scores of perceived knowledge, relevance and interest for each of the 33 geriatric topics/skills as well as overall knowledge mean score. Mean, SD, and mode of each topic knowledge, relevance and interest are reported in Supplementary Tables 5, 6, and 7, respectively.

Mean relevance and interest scores were consistently higher than self-reported knowledge across the majority of topics, suggesting a general perception of insufficient competence relative to clinical demands. Interestingly, mean knowledge score was always in the low-fair range and was comprised between 2.01 for the topic “assessing older people in ED” (lowest rank) to 3.55 for bone health (highest rank).

Conversely, both relevance and interest scores tended to be higher, generally falling in the *fair to high* category. This pattern was further supported by the analysis of score modes (Supplementary Tables 5, 6, and 7), which were most frequently *fair* for knowledge, but more commonly *high* for relevance and interest. Notably, *polypharmacy and deprescribing* consistently ranked at the top in both relevance and interest, whereas *assessing older people in the emergency department (ED)* ranked lowest in both domains, confirming the alignment of participants’ perceptions across these two dimensions.

#### Knowledge and competence

**Fig. 2:**
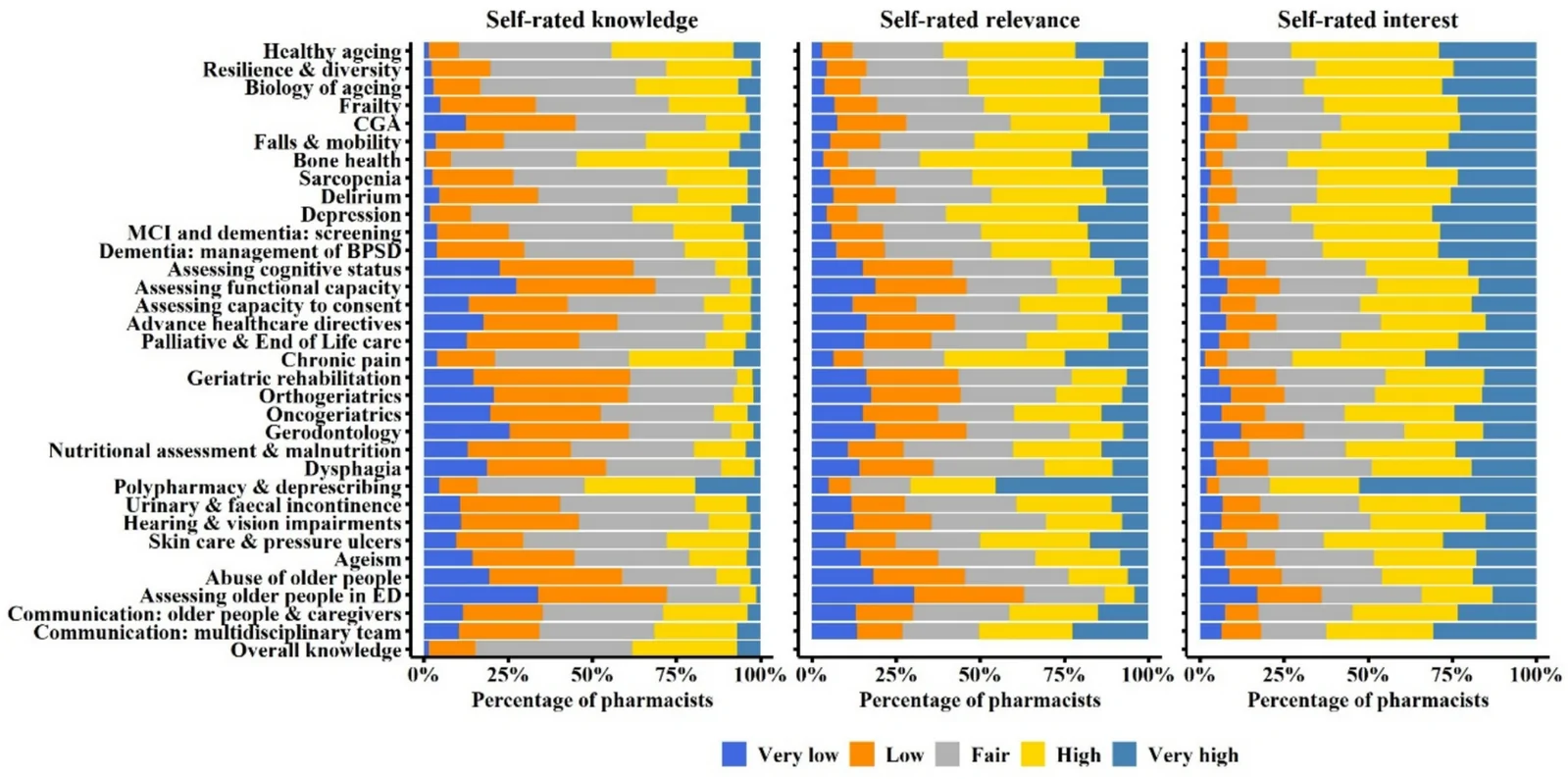
Pharmacists’ perceived knowledge, relevance and interest by topics or skills

Distribution of responses of participants in terms of perceived knowledge are reported in Fig. 2; the mean, median score, and modal responses for knowledge in each topic are detailed in Supplementary Table 5.

Across all topics and skills, the distribution of *self-rated knowledge* showed a clear shift towards the lower response categories, with relatively small proportions of professionals rating their knowledge as “very high”. In most domains, the majority of responses were clustered in the “fair” and “low” categories, while “very low” was more common in some topics including *assessing older people in ED*, *assessing functional capacity*, *assessing cognitive status*, and *gerodontology*. In contrast, topics such as *bone health*, *polypharmacy and deprescribing*, *healthy ageing*, and *depression* displayed higher proportions of “high” and “very high” responses.

These findings were consistent with the summary statistics from the Supplementary Table 4. *Bone health* and *polypharmacy & deprescribing* showed the highest mean knowledge scores (3.55 and 3.51, respectively), and a modal response of “high”. By contrast, several core topics, including *healthy ageing*, *depression*, *overall knowledge*, *biology of ageing*, *chronic pain*, *falls and mobility*, *resilience & diversity*, and *sarcopenia*, had mean scores just above 3.0 and a modal response of “fair”, suggesting an overall moderate perceived knowledge.

The lowest mean knowledge scores were instead observed for *assessing older people in ED* (mean = 2.01), *assessing functional capacity* (2.15), and *gerodontology* (2.24), with a median of 2 and a modal response of “low”. Similarly, low mean scores were seen for *orthogeriatrics* (2.29), *assessing cognitive status* (2.32), *geriatric rehabilitation* (2.33), and *abuse of older people* (2.38). For each of these topics, the predominant response category was “low”.

Overall, most topics fell in the *fair* knowledge range (median = 3, modal response = “fair”), indicating that while basic awareness is present, there is still substantial room for improvement across a broad set of geriatric competencies.

#### Relevance

Distribution of responses of participants in terms of perceived relevance are reported in Fig. 2; the mean, median score, and modal responses for relevance in each topic are detailed in Supplementary Table 6.

Overall, participants rated most geriatric topics as highly relevant to their clinical practice, with a clear tendency toward the upper response categories (“high” and “very high”). Compared with self-rated knowledge, the proportion of “very low” and “low” relevance responses was markedly smaller across all topics.

The most relevant topic was *polypharmacy and deprescribing*, with a mean score of 4 points and a mode score category of very high relevance. Other topics rated as the most relevant, with a higher proportion of high and very high categories were represented by *bone health*, *healthy ageing, chronic pain, depression, resilience and diversity*, *falls and mobility*, *sarcopenia*, *MCI and dementia screening,* all of which had mean relevance scores > 3.4 and a modal response of “high” (Supplementary Table 6). Intermediate relevance (mean between 3.2 and 3.4) was observed for *frailty*, *dementia and management of BPSD, communication in multidisciplinary teams, skin care & pression ulcers,* and *delirium*. These topics were generally rated as “highly relevant”, but the proportion of “very high” responses was more modest, reflected by a modal response of “high”, except for *dementia and management of BPSD* which had a mode of “fair”. A group of topics, including *CGA, nutritional assessment & malnutrition*, *communication with older people & caregivers*, *urinary and faecal incontinence*, *assessing capacity to consent*, *oncogeriatrics, palliative & end-of-life care*, *dysphagia*, *hearing and vision impairments*, *ageism* and *assessing cognitive status*, showed slightly lower mean relevance scores (~ 2.9 to 3.2), with “fair” being the most common response category. Finally, the lowest mean relevance ratings, in descending order, were reported for *assessing functional capacity* (mean = 2.71), *geriatric rehabilitation*, *gerodontology*, *abuse of older people*, *orthogeriatrics* and *assessing older people in ED* (2.24), with responses predominantly clustering in the “low” to “fair” categories.

#### Interest

Distribution of responses of participants in terms of perceived interest are reported in Fig. 2; the mean, median score, and modal responses for interest in each topic are detailed in Supplementary Table 7.

Compared to knowledge and relevance, self-rated *interest* displayed a generally favorable distribution, with the vast majority of participants reporting “high” or “very high” interest in most geriatric topics.

The highest level of interest was observed for *polypharmacy and deprescribing* (mean = 4.24; modal response = “very high”), in keeping with the large proportion of respondents reporting “very high” interest in this topic. Likewise, interest was particularly high for *bone health*, *depression*, *chronic pain*, and *healthy ageing* (mean scores ~ 3.9 to 4.0; modal response = “high”), suggesting substantial appetite for education in these core areas.

A second group of topics, including *biology of ageing*, *MCI and dementia screening*, *dementia management of BPSD*, *resilience & diversity*, *falls & mobility*, *delirium*, *sarcopenia*, *frailty*, *skin care & pressure ulcers* showed high mean interest scores (3.7–3.8) and a modal response of “high”, reflecting widespread enthusiasm for further training across a broad range of geriatric competencies.

Moderate but still predominantly high interest (mean 3.44–3.69) was seen for *communication in multidisciplinary teams, CGA, nutritional assessment*, *palliative & end-of-life care*, *oncogeriatrics*, *communication with older people and caregivers*, *urinary/faecal incontinence*, *assessing capacity to consent*, and *assessing cognitive status*. In these topics, “high” remained the most common response, although the proportion of “fair” responses was slightly larger.

A smaller subset of topics including *dysphagia*, *geriatric rehabilitation*, *abuse of older people*, *gerodontology*, and *assessing older people in ED*, were characterised by lower mean scores (2.94–3.43) and modal responses of “fair”.

### Identification of knowledge gaps and emerging priorities

**Fig. 3:**
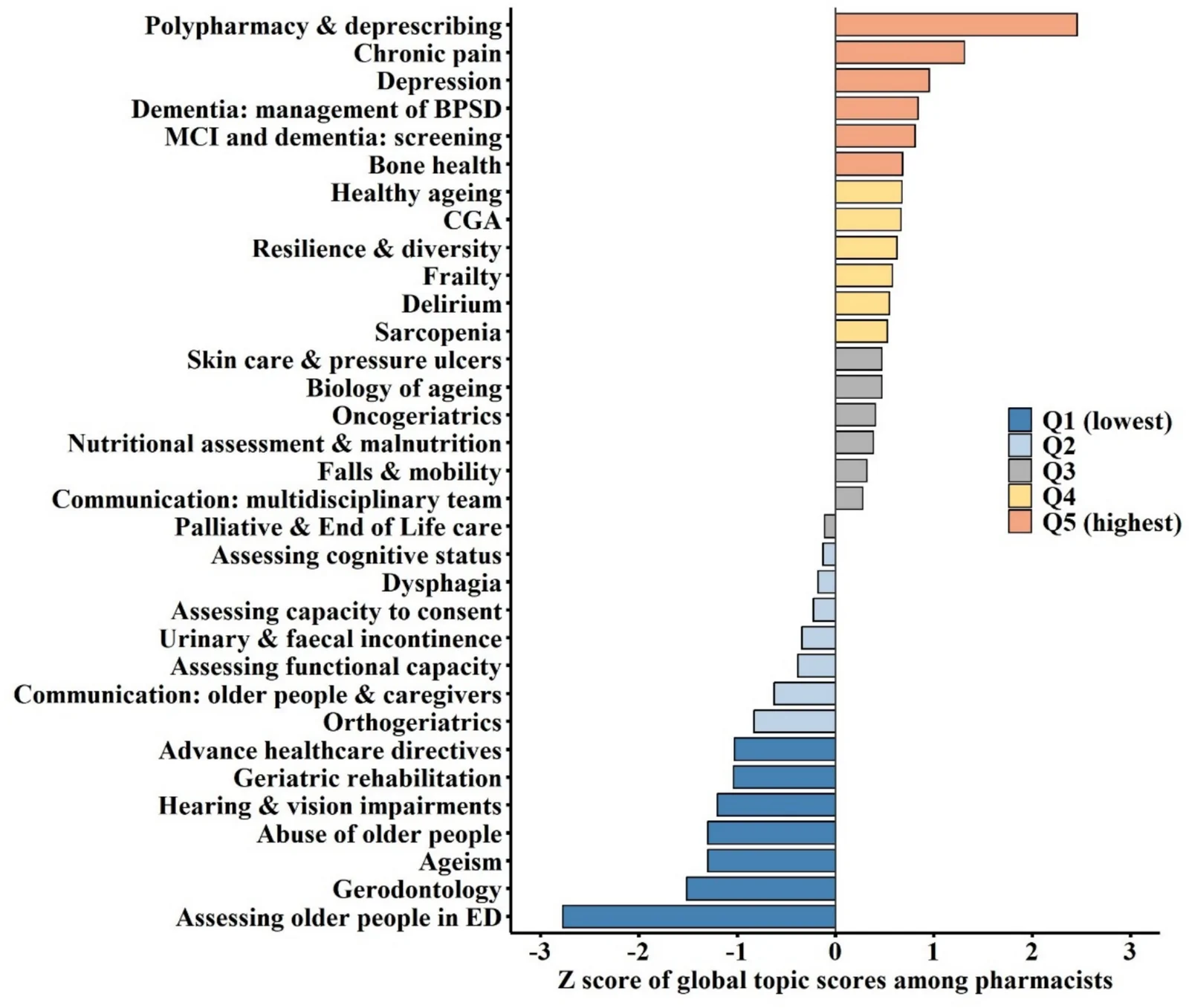
Standardized *Z*-scores of global topic scores

Figure 3 displays the standardized *Z*-scores of global topic scores (relevance + interest − knowledge) across surveyed domains of geriatric training. Topics falling in the highest quintiles (Q4–Q5) of *Z*-scores, shown in yellow and orange, represented knowledge gaps.

Among all topics, *polypharmacy and deprescribing, chronic pain, depression, management of BPSD, MCI and dementia screening*, and *bone health* were the top-ranked training priorities (green bars), all in quintile 5, with *Z*-scores ranging from 0.68 to 2.45; polypharmacy & deprescribing (*Z* score: 2.45) and chronic pain (*Z* score: 1.31) were the two topics with a *Z* score of global topic score > 1.

Other six topics, namely *healthy ageing, CGA, resilience and diversity, frailty, delirium*, and *sarcopenia* belonged to Q4 with *Z*-scores ranging from 0.53 to 0.67. In contrast, several topics fell in the lowest quintiles (Q1–Q2), with negative *Z*-scores reflecting lower overall training demand relative to the sample mean. Of note, some topics that clustered in the lowest quintiles were characterized by low-very low mean knowledge scores as well as by low modal knowledge category (Supplementary Table 5); these topics, including *assessing older people in ED* (having the worst knowledge, relevance and interest score), *gerodontology, abuse of older people*, and *advance healthcare directives¸* may represent emerging or under-recognized competencies in GM.

#### Prevalence of very low fair knowledge, relevance and interest scores according to age tertiles and sex

Supplementary Tables 8 to 13 summarize the prevalence of very low-fair knowledge, relevance, and interest categories across age tertiles and sex.

Distribution of such categories was substantially uniform and did not significantly change across groups.

## Discussion

Our survey assessed the interests and educational needs in geriatric medicine and older people care among 346 pharmacists, characterized by a broad geographic distribution of participants from more than 20 countries across Europe and beyond. We collected responses from pharmacists working in diverse settings, mostly in primary care or community, predominantly women. More than 80% of the participating pharmacists worked and have studied in one of the following countries: Albania, Türkiye, Croatia and Serbia.

### Exposure and interest in GM courses

Formal exposure to GM during undergraduate or postgraduate education was generally limited, with only one in three respondents reporting attendance at dedicated courses. Practical experiences such as clinical rotations in geriatric wards, rehabilitation units, or nursing homes were even less common, each reported by less than 10% of pharmacists. While more than 40% of respondents reported high or very high enjoyment in engaging with older people, a much larger proportion (over four in five) rated the social prestige of caring for older adults as “very little” or “fairly” prestigious. This contrast suggests that pharmacists’ motivation to work with older patients is driven more by personal or professional values than by societal recognition.

Our survey also shed light on the barriers pharmacists face in pursuing geriatric training. The most frequently reported obstacles were time constraints, lack of paid leave, and concerns about financial loss, each affecting a substantial proportion of respondents. Additional barriers included doubts about the feasibility or relevance of the taught skills and, to a lesser extent, language difficulties when courses were offered in English. Only a minority reported low motivation to attend, suggesting that willingness to engage in geriatric education is generally high, but structural and contextual factors hinder participation. Age-related patterns added further nuance. Indeed, younger participants cited financial and time constraints, whereas older pharmacists more often reported language barriers and doubts about the necessity of training. These findings may suggest the need for diversified strategies to support the uptake of geriatric training, ranging from structured and financially supported programs for early-career pharmacists, to flexible opportunities in native languages for mid to late-career professionals.

### Knowledge gaps and emerging priorities

The second most interesting finding of our study was the modest self-rated knowledge across all topics, clustering around the low-to-fair range, while both perceived relevance and interest were consistently higher. This imbalance reflects a widespread recognition of the importance of geriatric topics alongside perceived gaps in competence. Polypharmacy and deprescribing, bone health, depression, chronic pain, and healthy ageing consistently ranked highest across knowledge, relevance, and interest, underscoring their centrality to clinical pharmacy practice in ageing populations. When training priorities were quantified using standardized *Z*-scores of global topic scores (relevance + interest − knowledge), polypharmacy and deprescribing emerged as the strongest educational need, followed by chronic pain, depression, dementia screening and BPSD management, and bone health. Other important areas included frailty, delirium, sarcopenia, resilience and diversity, and comprehensive geriatric assessment.

Evidence from studies conducted in Malaysia, Canada, China, and Saudi Arabia suggests that pharmacists’ preparedness for geriatric care varies substantially across domains. While Abd Wahab et al. reported overall insufficient knowledge and gaps in formal geriatric pharmacotherapy education, Allen et al. identified lower confidence in frailty, cognitive, and functional assessment, and Hu et al. demonstrated favourable knowledge and practices primarily related to polypharmacy management, indicating stronger competence in medication-focused tasks [15, 18, 19]. Similarly, studies addressing pain management found inadequate knowledge among community and frontline pharmacists [20, 21]. Collectively, these findings point to persistent, domain-specific educational gaps extending beyond pharmacotherapy optimisation alone.

It is of note that some topics that clustered in the lowest quintiles (Q1–Q2) of *Z*-scores were characterized by low-very low mean knowledge scores as well as by low modal knowledge category; these topics included *assessing older people in ED (*having the lowest knowledge, relevance and interest score), *gerodontology, abuse of older people*, and *advance healthcare directives.* From an educational perspective, these areas are not perceived as immediate training priorities by respondents, since both the perceived need (relevance, interest) and the perceived knowledge gaps are limited. In the context of shaping geriatric curricula, such findings suggest that these domains may be less emphasized in targeted training programs, particularly when resources are limited and must be focused on high-priority areas (e.g., polypharmacy, dementia, frailty). However, it is also important to note that low scores do not necessarily imply lack of importance for geriatric practice. Instead, they may reflect a) limited awareness of their clinical or societal relevance (e.g., gerodontology, abuse of older people); b) contextual differences in clinical practice across settings (e.g., ED assessment of older adults), or c) overlap with other domains that participants considered more directly relevant (e.g., frailty vs. geriatric rehabilitation). Thus, while these topics may not currently drive training demand, they could represent emerging or under-recognized competencies that warrant careful consideration in comprehensive curricula.

In our study, gender and age differences in pharmacists’ exposure to GM training were modest and largely non-significant, aligning with limited and inconsistent evidence from prior research on demographic influences in geriatric pharmacotherapy [15]. The sole significant gender finding was lower participation of women in acute geriatric ward rotations (6.7% vs. 15.0% in men, *p* = 0.047), potentially indicating unequal access during education or early practice, though no differences emerged in formal GM course attendance, interest in GM education, training barriers, or distribution of self-rated knowledge, relevance, and interest across topics (Supplementary Tables 8–13). This contrasts with some external studies where female pharmacists showed higher deprescribing readiness in community settings [22], but echoes others reporting no gender differences in knowledge or attitudes toward older adults [15].

Age tertile stratification (23–30, 31–42, 43–72 years) revealed non-significant trends: younger pharmacists had slightly lower formal GM course attendance (29.2% vs. 35.6% in the oldest) but marginally higher practical rotation exposure (e.g., 9.2% vs. 5.2% in acute wards). Interest in GM courses increased modestly with age (46.9% in youngest vs. 56.5% in oldest, *p* = 0.305), with significant differences in format preferences (*p* = 0.022; younger favoring hybrid/online, older in-person) and barriers (younger citing financial/time/paid leave issues and skill feasibility doubts; older noting language barriers and doubts about skill necessity). However, very low-to-fair knowledge, relevance, and interest scores remained uniformly distributed across age groups for individual topics, with no evidence of differential prioritization (e.g., no age-specific preferences for polypharmacy/deprescribing vs. frailty/chronic pain). This supports findings from other cohorts where experience (> 10 years, proxy for age) linked to higher polypharmacy knowledge/practice [23], but demographic factors overall appear weaker than curricular gaps, targeted education, and institutional support in shaping geriatric competence.

These results underscore the need for equitable access to geriatric rotations (particularly for women) and age-tailored continuing education to address persistent training deficiencies in core areas like polypharmacy, deprescribing, frailty, and chronic pain.

Our study has many strengths. To our knowledge, this is the first open online survey exploring pharmacists´ perceived educational needs and attitudes towards geriatric medicine that was conducted in multiple languages and harmonized across countries primarily in Europe. The survey was anonymous so to allow respondents to freely express their knowledge gaps, educational needs and perceived barriers against attending geriatric medicine courses, thus reducing social desirability bias. The online format allowed participation from countries across Europe and beyond, capturing diverse perspectives and increasing the external validity of the findings. Further strengths include the large number of respondents, the inclusion of participants of different ages, previous education and current work in different countries, and the richness of topics covered by the survey. Although certification modalities were out of scope of PROGRAMMING COST Action per se, the gaps highlighted in our findings, particularly in areas related to polypharmacy, deprescribing, and complex geriatric syndromes, may help inform competency-based educational initiatives and future certification models.

Nevertheless, some of the characteristics that enabled broad international participation also introduce limitations that should be considered when interpreting the findings. A potential limitation of our study is the unequal distribution of respondents across the participating countries. The participation was voluntary, which may have led to overrepresentation of pharmacists already interested in geriatrics or those more engaged professionally, therefore pharmacists less familiar with or less interested in geriatrics may be underrepresented. Furthermore, as the questionnaire was designed for an interprofessional audience, some competencies, particularly those related to standardized clinical assessment, such as assessing older people in the emergency department, functional capacity, and cognitive status may have been perceived as less directly aligned with pharmacists’ usual scope of practice, which could have influenced the low ratings in these domains. However, the inclusion of some aspects of these topics in pharmacists’ curricula could be considered: in many cases pharmacists are the first-line healthcare professionals encountering older people in the community, who face limited access to the whole scope of care services. In this context, increased awareness and clinical suspicion of conditions such as still undiagnosed cognitive problems and underlying functional decline may be of particular relevance for medication self-management and patient safety aspects and pharmacists stand at a strategic place to identify these conditions and refer patients accordingly.

One section of the survey explored interest in courses in GM and focused on various types of educational delivery. However, we acknowledge that the development of an educational program in geriatric medicine should be co-designed by multiple stakeholders through a rigorous process as described in previous literature [24, 25]. Moreover, the large age range of our study participants may impact on our findings on the preferred types of educational delivery.

### Future implications

The educational priorities identified in this survey may have implications for the development of structured training and certification pathways in geriatric pharmacy across Europe, where postgraduate training and certification opportunities in GM for pharmacists remain heterogeneous across countries.

Leveraging existing CPD requirements could represent a practical strategy to strengthen geriatric competencies, by integrating targeted training modules aligned with identified learning needs and including pharmacists in a pluri-professional team educational model. Such approaches may support harmonization of geriatric education while reinforcing pharmacists’ evolving role within multidisciplinary care for older adults. Further research on implementation and evaluation of targeted professional education and training programs should explore whether these programs on basic geriatric competencies may meet the educational needs of pharmacists and result in meaningful clinical outcomes for the improvement of the quality of care of older people.

## Supplementary Information

Below is the link to the electronic supplementary material.

Supplementary file1 (DOCX 81 kb)

## Data Availability

The data that support the fi ndings of this study are available from the corresponding author upon reasonable request.
