# Supplementary material for: Pharmacists’ perceived knowledge, attitudes and practice in geriatrics: findings from a multinational multilingual open online survey
Source: Eur Geriatr Med. 2026 May 14;17(4):1857–71. doi: 10.1007/s41999-026-01504-z (PMC13562129; doi:10.1007/s41999-026-01504-z)
Supplement: Supplementary file 1 [file 41999_2026_1504_MOESM1_ESM.docx]

**Supplementary table 1.** Previous Attendance of courses in GM in the whole study population and among men and women

|  | | **All (n=346)** | **Men (n=60)** | **Women (n=285)** | **P value** |
| --- | --- | --- | --- | --- | --- |
| Attended courses in GM | |  |  |  | 0.483 |
|  | Yes | 112 (32.4) | 18 (30.0) | 93 (33.0) |  |
|  | No | 209 (60.4) | 35 (58.3) | 73 (60.7) |  |
|  | Not sure | 25 (7.2) | 7 (11.7) | 18 (6.3) |  |
| Rotations in GM acute hospital ward | |  |  |  | **0.047** |
|  | Yes | 28 (8.1) | 9 (15.0) | 19 (6.7) |  |
|  | No | 299 (86.4) | 45 (75.0) | 253 (88.8) |  |
|  | Not sure | 19 (5.5) | 6 (10.0) | 3 (4.6) |  |
| Rotations in rehabilitation setting, n (%) | |  |  |  | 0.138 |
|  | Yes | 7 (2.0) | 3 (5.0) | 4 (1.4) |  |
|  | No | 321 (92.8) | 52 (86.7) | 268 (94.0) |  |
|  | Not sure | 18 (5.2) | 5 (8.3) | 13 (4.6) |  |
| Rotations in care home or nursing home, n (%) | |  |  |  | 0.132 |
|  | Yes | 15 (4.3) | 6 (10.0) | 9 (3.2) |  |
|  | No | 314 (90.8) | 50 (83.3) | 263 (92.3) |  |
|  | Not sure | 17 (4.9) | 4 (6.7) | 13 (4.6) |  |
| Rotations in outpatient clinic, n (%) | |  |  |  | 0.138 |
|  | Yes | 22 (6.4) | 7 (11.7) | 15 (5.3) |  |
|  | No | 308 (89.0) | 48 (80.0) | 259 (90.9) |  |
|  | Not sure | 16 (4.6) | 5 (8.3) | 11 (3.9) |  |
| Volunteer work, n (%) | |  |  |  | 0.186 |
|  | Yes | 76 (22.0) | 12 (20.0) | 64 (22.5) |  |
|  | No | 258 (74.6) | 43 (71.7) | 214 (75.1) |  |
|  | Not sure | 12 (3.4) | 5 (8.3) | 7 (2.5) |  |
| Research work, n (%) | |  |  |  | 0.568 |
|  | Yes | 79 (22.8) | 10 (16.7) | 69 (24.2) |  |
|  | No | 254 (73.4) | 47 (78.3) | 206 (72.3) |  |
|  | Not sure | 13 (3.8) | 3 (5.0) | 10 (3.5) |  |

**Supplementary Table 2**. Interest in care of older people in the whole study population and across age tertiles

|  | | **Age tertiles** | | | | |
| --- | --- | --- | --- | --- | --- | --- |
|  | | **All (n=346)** | **23-30 years (n=130)** | **31-42 years (n=101)** | **43-72 years (n=115)** | **P value** |
| Enjoy engaging with older people, n (%) | |  |  |  |  | 0.325 |
|  | Very low-fair | 203 (58.7) | 80 (61.5) | 62 (61.4) | 61 (53.0) |  |
|  | High-very high | 143 (41.3) | 50 (38.5) | 39 (38.6) | 54 (47.0) |  |
| Prestigious caring for older people in your country, n (%) | |  |  |  |  | 0.155 |
|  | Very little-Fairly | 291 (84.1) | 106 (81.5) | 86 (85.1) | 99 (86.0) |  |
|  | Much-Very much | 40 (11.6) | 20 (15.4) | 12 (11.9) | 8 (7.0) |  |
|  | Not sure | 15 (4.3) | 4 (3.1) | 3 (3.0) | 8 (7.0) |  |

**Supplementary Table 3.** Interest in care of older people and courses in GM in the whole study population and among men and women.

|  | | **All (n=346*)** | **Men (n=60)** | **Women (n=285)** | **P value** |
| --- | --- | --- | --- | --- | --- |
| Enjoy engaging older people, n (%) | |  |  |  | 0.191 |
|  | Very low-fair | 203 (58.7) | 30 (50.0) | 172 (60.4) |  |
|  | High-very high | 143 (41.3) | 30 (50.0) | 113 (39.6) |  |
| Prestigious caring for older people in your country, n (%) | |  |  |  | 0.729 |
|  | Very little-Fairly | 291 (84.1) | 52 (86.7) | 238 (83.5) |  |
|  | Much-Too much | 40 (11.6) | 7 (11.7) | 33 (11.6) |  |
|  | Not sure | 15 (4.3) | 1 (1.7) | 14 (4.9) |  |

***1 answering “not sure” to the gender question was removed from the table.**

**Supplementary Table 4.** Interest in courses in GM in the whole study population and among men and women.

|  | **All (n=346*)** | **Men (n=60)** | **Women (n=285)** | **P value** |
| --- | --- | --- | --- | --- |
| Interest in attending courses in GM, n (%) |  |  |  | 0.721 |
| Very little-Fairly | 166 (48.0) | 27 (45.0) | 139 (48.8) |  |
| Much-Too much | 180 (52.0) | 33 (55.0) | 146 (51.2) |  |
| Which type of course, n (%) |  |  |  | 0.137 |
| In-person | 44 (12.7) | 12 (20.0) | 22 (11.2) |  |
| Online | 131 (37.9) | 17 (28.3) | 114 (40.0) |  |
| Hybrid | 171 (49.4) | 31 (51.7) | 48 (48.8) |  |
| *Barriers, n (%)* |  |  |  |  |
| Financial loss |  |  |  | 0.993 |
| Yes | 139 (40.2) | 24 (40.0) | 115 (40.4) |  |
| No | 138 (39.9) | 24 (40.0) | 113 (39.6) |  |
| Not sure | 69 (19.9) | 12 (20.0) | 57 (20.0) |  |
| Paid leave issues |  |  |  | 0.191 |
| Yes | 143 (41.3) | 31 (51.7) | 112 (39.3) |  |
| No | 140 (40.5) | 22 (36.7) | 117 (41.1) |  |
| Not sure | 63 (18.2) | 56 (19.6) | 0 |  |
| Doubts about feasibility of taught skills |  |  |  | 0.752 |
| Yes | 97 (28.0) | 18 (30.0) | 79 (27.7) |  |
| No | 177 (51.2) | 27 (45.0) | 149 (52.3) |  |
| Not sure | 72 (20.8) | 15 (25.0) | 57 (20.0) |  |
| Doubts about the relevance of taught skills |  |  |  | 0.945 |
| Yes | 68 (19.7) | 13 (21.7) | 55 (19.3) |  |
| No | 223 (64.4) | 38 (63.3) | 184 (64.6) |  |
| Not sure | 55 (15.9) | 9 (15.0) | 46 (16.1) |  |
| Doubts about the necessity to improve skills |  |  |  | **0.636** |
| Yes | 47 (13.6) | 11 (18.3) | 36 (12.6) |  |
| No | 250 (72.2) | 41 (68.3) | 208 (73.0) |  |
| Not sure | 49 (14.2) | 8 (13.3) | 41 (14.4) |  |
| Language barrier |  |  |  | 0.132 |
| Yes | 65 (18.8) | 7 (11.7) | 57 (20.0) |  |
| No | 237 (68.5) | 43 (71.7) | 194 (68.1) |  |
| Not sure | 44 (12.7) | 10 (16.7) | 34 (11.9) |  |
| Low motivation |  |  |  | 0.294 |
| Yes | 40 (11.6) | 9 (15.0) | 31 (10.9) |  |
| No | 260 (75.1) | 44 (73.3) | 216 (75.8) |  |
| Not sure | 46 (13.3) | 7 (11.7) | 38 (13.3) |  |
| Time constraints |  |  |  | 0.615 |
| Yes | 218 (63.0) | 38 (63.3) | 180 (63.2) |  |
| No | 85 (24.6) | 15 (25.0) | 69 (24.2) |  |
| Not sure | 43 (12.4) | 7 (11.7) | 36 (12.6) |  |

***1 answering “not sure” to the gender question was removed from the table.**

**Supplementary Table 5.** Pharmacists’ perceived knowledge: distribution of responses by descending mean score

| **Topics and skills** | **Knowledge mean score** | **Knowledge median score** | **Knowledge modal response** |
| --- | --- | --- | --- |
| Bone health | 3.55 | 4 | 4-high |
| Polypharmacy & deprescribing | 3.51 | 4 | 4-high |
| Healthy ageing | 3.40 | 3 | 3-fair |
| Depression | 3.31 | 3 | 3-fair |
| Overall knowledge | 3.28 | 3 | 3-fair |
| Biology of ageing | 3.24 | 3 | 3-fair |
| Chronic pain | 3.22 | 3 | 3-fair |
| Falls & mobility | 3.13 | 3 | 3-fair |
| Resilience & diversity | 3.09 | 3 | 3-fair |
| Sarcopenia | 3.02 | 3 | 3-fair |
| MCI and dementia: screening | 3.01 | 3 | 3-fair |
| Communication: multidisciplinary team | 2.94 | 3 | 3-fair |
| Frailty | 2.93 | 3 | 3-fair |
| Dementia: management of BPSD | 2.93 | 3 | 3-fair |
| Skin care & pressure ulcers | 2.92 | 3 | 3-fair |
| Delirium | 2.90 | 3 | 3-fair |
| Communication: older people & caregivers | 2.86 | 3 | 3-fair |
| Urinary & faecal incontinence | 2.72 | 3 | 3-fair |
| Nutritional assessment & malnutrition | 2.67 | 3 | 3-fair |
| Ageism | 2.66 | 3 | 3-fair |
| Assessing capacity to consent | 2.64 | 3 | 3-fair |
| CGA | 2.62 | 3 | 3-fair |
| Palliative & End of Life care | 2.62 | 3 | 3-fair |
| Hearing & vision impairments | 2.61 | 3 | 3-fair |
| Oncogeriatrics | 2.45 | 2 | 3-fair |
| Dysphagia | 2.40 | 2 | 2-low |
| Advance healthcare directives | 2.38 | 2 | 2-low |
| Abuse of older people | 2.38 | 2 | 2-low |
| Geriatric rehabilitation | 2.33 | 2 | 2-low |
| Assessing cognitive status | 2.32 | 2 | 2-low |
| Orthogeriatrics | 2.29 | 2 | 2-low |
| Gerodontology | 2.24 | 2 | 2-low |
| Assessing functional capacity | 2.15 | 2 | 2-low |
| Assessing older people in ED | 2.01 | 2 | 2-low |

Abbreviations: CGA: Comprehensive Geriatric Assessment; MCI: mild cognitive impairment; BPSD: behavioural and psychiatric symptoms of dementia; ED: Emergency Department.

**Supplementary Table 6.** Pharmacists’ perceived relevance: distribution of responses by descending mean score

| **Topics and skills** | **Relevance mean score** | **Relevance median score** | **Relevance modal response** |
| --- | --- | --- | --- |
| Polypharmacy & deprescribing | 4.00 | 4.0 | 5-very high |
| Bone health | 3.77 | 4.0 | 4-high |
| Healthy ageing | 3.67 | 4.0 | 4-high |
| Chronic pain | 3.64 | 4.0 | 4-high |
| Depression | 3.63 | 4.0 | 4-high |
| Biology of ageing | 3.50 | 4.0 | 4-high |
| Resilience & diversity | 3.46 | 4.0 | 4-high |
| Falls & mobility | 3.44 | 4.0 | 4-high |
| Sarcopenia | 3.42 | 4.0 | 4-high |
| MCI and dementia: screening | 3.41 | 3.0 | 4-high |
| Frailty | 3.37 | 3.0 | 4-high |
| Dementia: management of BPSD | 3.35 | 3.0 | 3-fair |
| Communication: multidisciplinary team | 3.33 | 4.0 | 4-high |
| Skin care & pressure ulcers | 3.32 | 3.5 | 4-high |
| Delirium | 3.28 | 3.0 | 4-high |
| CGA | 3.17 | 3.0 | 3-fair |
| Nutritional assessment & malnutrition | 3.16 | 3.0 | 3-fair |
| Communication: older people & caregivers | 3.13 | 3.0 | 3-fair |
| Urinary & faecal incontinence | 3.11 | 3.0 | 3-fair |
| Assessing capacity to consent | 3.07 | 3.0 | 3-fair |
| Oncogeriatrics | 3.01 | 3.0 | 4-high |
| Palliative & End of Life care | 2.97 | 3.0 | 3-fair |
| Dysphagia | 2.91 | 3.0 | 3-fair |
| Hearing & vision impairments | 2.90 | 3.0 | 3-fair |
| Ageism | 2.90 | 3.0 | 3-fair |
| Assessing cognitive status | 2.82 | 3.0 | 3-fair |
| Advance healthcare directives | 2.76 | 3.0 | 3-fair |
| Orthogeriatrics | 2.73 | 3.0 | 3-fair |
| Assessing functional capacity | 2.70 | 3.0 | 2-low |
| Geriatric rehabilitation | 2.69 | 3.0 | 3-fair |
| Gerodontology | 2.66 | 3.0 | 3-fair |
| Abuse of older people | 2.66 | 3.0 | 3-fair |
| Assessing older people in ED | 2.24 | 2.0 | 2-low |

Abbreviations: CGA: Comprehensive Geriatric Assessment; MCI: mild cognitive impairment; BPSD: behavioural and psychiatric symptoms of dementia; ED: Emergency Department.

**Supplementary Table 7.** Pharmacists’ perceived interest: distribution of responses by descending mean score

| **Topics and skills** | **Interest mean score** | **Interest median score** | **Interest modal response** |
| --- | --- | --- | --- |
| Polypharmacy & deprescribing | 4.24 | 5 | 5-very high |
| Bone health | 3.98 | 4 | 4-high |
| Depression | 3.96 | 4 | 4-high |
| Chronic pain | 3.96 | 4 | 4-high |
| Healthy ageing | 3.92 | 4 | 4-high |
| Biology of ageing | 3.88 | 4 | 4-high |
| MCI and dementia: screening | 3.84 | 4 | 4-high |
| Dementia: management of BPSD | 3.82 | 4 | 4-high |
| Resilience & diversity | 3.80 | 4 | 4-high |
| Falls & mobility | 3.78 | 4 | 4-high |
| Delirium | 3.78 | 4 | 4-high |
| Sarcopenia | 3.76 | 4 | 4-high |
| Frailty | 3.73 | 4 | 4-high |
| Skin care & pressure ulcers | 3.73 | 4 | 4-high |
| Communication: multidisciplinary team | 3.68 | 4 | 4-high |
| CGA | 3.64 | 4 | 4-high |
| Nutritional assessment & malnutrition | 3.62 | 4 | 4-high |
| Palliative & End of Life care | 3.61 | 4 | 4-high |
| Oncogeriatrics | 3.55 | 4 | 4-high |
| Communication: older people & caregivers | 3.53 | 4 | 4-high |
| Urinary & faecal incontinence | 3.51 | 4 | 4-high |
| Assessing capacity to consent | 3.49 | 4 | 4-high |
| Assessing cognitive status | 3.45 | 4 | 4-high |
| Dysphagia | 3.43 | 3 | 3-fair |
| Ageism | 3.36 | 3 | 4-high |
| Hearing & vision impairments | 3.34 | 3 | 4-high |
| Assessing functional capacity | 3.33 | 3 | 4-high |
| Geriatric rehabilitation | 3.32 | 3 | 3-fair |
| Abuse of older people | 3.32 | 3 | 3-fair |
| Advance healthcare directives | 3.31 | 3 | 4-high |
| Orthogeriatrics | 3.30 | 3 | 4-high |
| Gerodontology | 3.12 | 3 | 3-fair |
| Assessing older people in ED | 2.94 | 3 | 3-fair |

Abbreviations: CGA: Comprehensive Geriatric Assessment; MCI: mild cognitive impairment; BPSD: behavioural and psychiatric symptoms of dementia; ED: Emergency Department.

**Supplementary Table 8.** Distribution of very low-fair perceived knowledge of topics and skills across age tertiles

| **Topic, n (%)** | **All (n=346)** | **23-30 years (n=130)** | **31-42 years (n=101)** | **43-72 years (n=115)** | **P value** |
| --- | --- | --- | --- | --- | --- |
| Depression | 215 (62.1%) | 74 (56.9%) | 63 (62.4%) | 78 (67.8%) | 0.21 |
| MCI and dementia: screening | 257 (74.3%) | 93 (71.5%) | 78 (77.2%) | 86 (74.8%) | 0.61 |
| Dementia: management of BPSD | 268 (77.5%) | 96 (73.8%) | 82 (81.2%) | 90 (78.3%) | 0.40 |
| Assessing cognitive status | 300 (86.7%) | 112 (86.2%) | 88 (87.1%) | 100 (87%) | 0.97 |
| Assessing functional capacity | 315 (91%) | 117 (90%) | 90 (89.1%) | 108 (93.9%) | 0.41 |
| Assessing capacity to consent | 288 (83.2%) | 109 (83.8%) | 86 (85.1%) | 93 (80.9%) | 0.68 |
| Advance healthcare directives | 308 (89%) | 117 (90%) | 88 (87.1%) | 103 (89.6%) | 0.77 |
| Palliative & End of Life care | 290 (83.8%) | 109 (83.8%) | 85 (84.2%) | 96 (83.5%) | 0.99 |
| Chronic pain | 211 (61%) | 80 (61.5%) | 59 (58.4%) | 72 (62.6%) | 0.81 |
| Geriatric rehabilitation | 322 (93.1%) | 122 (93.8%) | 94 (93.1%) | 106 (92.2%) | 0.88 |
| Healthy ageing | 193 (55.8%) | 77 (59.2%) | 58 (57.4%) | 58 (50.4%) | 0.36 |
| Orthogeriatrics | 318 (91.9%) | 118 (90.8%) | 91 (90.1%) | 109 (94.8%) | 0.38 |
| Oncogeriatrics | 298 (86.1%) | 110 (84.6%) | 86 (85.1%) | 102 (88.7%) | 0.62 |
| Gerodontology | 316 (91.3%) | 115 (88.5%) | 94 (93.1%) | 107 (93%) | 0.34 |
| Nutritional assessment & malnutrition | 278 (80.3%) | 101 (77.7%) | 81 (80.2%) | 96 (83.5%) | 0.52 |
| Dysphagia | 306 (88.4%) | 115 (88.5%) | 85 (84.2%) | 106 (92.2%) | 0.18 |
| Polypharmacy & deprescribing | 165 (47.7%) | 59 (45.4%) | 47 (46.5%) | 59 (51.3%) | 0.63 |
| Urinary & faecal incontinence | 279 (80.6%) | 107 (82.3%) | 81 (80.2%) | 91 (79.1%) | 0.81 |
| Hearing & vision impairments | 293 (84.7%) | 109 (83.8%) | 84 (83.2%) | 100 (87%) | 0.70 |
| Skin care & pressure ulcers | 250 (72.3%) | 89 (68.5%) | 77 (76.2%) | 84 (73%) | 0.41 |
| Ageism | 273 (78.9%) | 99 (76.2%) | 86 (85.1%) | 88 (76.5%) | 0.19 |
| Resilience & diversity | 249 (72%) | 101 (77.7%) | 72 (71.3%) | 76 (66.1%) | 0.13 |
| Abuse of older people | 301 (87%) | 113 (86.9%) | 89 (88.1%) | 99 (86.1%) | 0.91 |
| Assessing older people in ED | 325 (93.9%) | 120 (92.3%) | 93 (92.1%) | 112 (97.4%) | 0.16 |
| Communication: older people & caregivers | 246 (71.1%) | 91 (70%) | 70 (69.3%) | 85 (73.9%) | 0.71 |
| Communication: multidisciplinary team | 237 (68.5%) | 91 (70%) | 62 (61.4%) | 84 (73%) | 0.16 |
| Overall knowledge | 214 (61.8%) | 80 (61.5%) | 65 (64.4%) | 69 (60%) | 0.80 |
| Biology of ageing | 218 (63%) | 76 (58.5%) | 63 (62.4%) | 79 (68.7%) | 0.25 |
| Frailty | 252 (72.8%) | 98 (75.4%) | 75 (74.3%) | 79 (68.7%) | 0.47 |
| CGA | 290 (83.8%) | 113 (86.9%) | 83 (82.2%) | 94 (81.7%) | 0.47 |
| Falls & mobility | 228 (65.9%) | 84 (64.6%) | 64 (63.4%) | 80 (69.6%) | 0.59 |
| Bone health | 157 (45.4%) | 50 (38.5%) | 45 (44.6%) | 62 (53.9%) | ***0.05*** |
| Sarcopenia | 250 (72.3%) | 96 (73.8%) | 68 (67.3%) | 86 (74.8%) | 0.42 |
| Delirium | 261 (75.4%) | 91 (70%) | 78 (77.2%) | 92 (80%) | 0.17 |

Abbreviations: CGA: Comprehensive Geriatric Assessment; MCI: mild cognitive impairment; BPSD: behavioural and psychiatric symptoms of dementia; ED: Emergency Department.

**Supplementary Table 9.** Distribution of very low-fair perceived knowledge of topics and skills in the overall population and among men and women

| **Topic, n (%)** | **Overall (n=346)** | **Men (n=80)** | **Women (n=245)** | **P value** |
| --- | --- | --- | --- | --- |
| Depression | 214 (62%) | 35 (58.3%) | 179 (62.8%) | 0.62 |
| MCI and dementia: screening | 256 (74.2%) | 41 (68.3%) | 215 (75.4%) | 0.33 |
| Dementia: management of BPSD | 267 (77.4%) | 44 (73.3%) | 223 (78.2%) | 0.51 |
| Assessing cognitive status | 299 (86.7%) | 50 (83.3%) | 249 (87.4%) | 0.53 |
| Assessing functional capacity | 314 (91%) | 49 (81.7%) | 265 (93%) | 0.01 |
| Assessing capacity to consent | 287 (83.2%) | 46 (76.7%) | 241 (84.6%) | 0.19 |
| Advance healthcare directives | 307 (89%) | 51 (85%) | 256 (89.8%) | 0.39 |
| Palliative & End of Life care | 289 (83.8%) | 47 (78.3%) | 242 (84.9%) | 0.29 |
| Chronic pain | 210 (60.9%) | 30 (50%) | 180 (63.2%) | 0.08 |
| Geriatric rehabilitation | 321 (93%) | 53 (88.3%) | 268 (94%) | 0.19 |
| Healthy ageing | 192 (55.7%) | 36 (60%) | 156 (54.7%) | 0.55 |
| Orthogeriatrics | 317 (91.9%) | 52 (86.7%) | 265 (93%) | 0.17 |
| Oncogeriatrics | 297 (86.1%) | 50 (83.3%) | 247 (86.7%) | 0.64 |
| Gerodontology | 315 (91.3%) | 54 (90%) | 261 (91.6%) | 0.89 |
| Nutritional assessment & malnutrition | 277 (80.3%) | 46 (76.7%) | 231 (81.1%) | 0.55 |
| Dysphagia | 305 (88.4%) | 48 (80%) | 257 (90.2%) | 0.04 |
| Polypharmacy & deprescribing | 164 (47.5%) | 26 (43.3%) | 138 (48.4%) | 0.57 |
| Urinary & faecal incontinence | 278 (80.6%) | 43 (71.7%) | 235 (82.5%) | 0.08 |
| Hearing & vision impairments | 292 (84.6%) | 49 (81.7%) | 243 (85.3%) | 0.61 |
| Skin care & pressure ulcers | 249 (72.2%) | 42 (70%) | 207 (72.6%) | 0.80 |
| Ageism | 272 (78.8%) | 43 (71.7%) | 229 (80.4%) | 0.19 |
| Resilience & diversity | 248 (71.9%) | 41 (68.3%) | 207 (72.6%) | 0.61 |
| Abuse of older people | 300 (87%) | 50 (83.3%) | 250 (87.7%) | 0.48 |
| Assessing older people in ED | 324 (93.9%) | 56 (93.3%) | 268 (94%) | 1.00 |
| Communication: older people & caregivers | 245 (71%) | 43 (71.7%) | 202 (70.9%) | 1.00 |
| Communication: multidisciplinary team | 236 (68.4%) | 38 (63.3%) | 198 (69.5%) | 0.44 |
| Overall knowledge | 213 (61.7%) | 35 (58.3%) | 178 (62.5%) | 0.65 |
| Biology of ageing | 217 (62.9%) | 34 (56.7%) | 183 (64.2%) | 0.34 |
| Frailty | 251 (72.8%) | 38 (63.3%) | 213 (74.7%) | 0.10 |
| CGA | 289 (83.8%) | 47 (78.3%) | 242 (84.9%) | 0.29 |
| Falls & mobility | 227 (65.8%) | 40 (66.7%) | 187 (65.6%) | 0.99 |
| Bone health | 156 (45.2%) | 22 (36.7%) | 134 (47%) | 0.19 |
| Sarcopenia | 249 (72.2%) | 38 (63.3%) | 211 (74%) | 0.13 |
| Delirium | 260 (75.4%) | 41 (68.3%) | 219 (76.8%) | 0.22 |

Abbreviations: CGA: Comprehensive Geriatric Assessment; MCI: mild cognitive impairment; BPSD: behavioural and psychiatric symptoms of dementia; ED: Emergency Department.

**Supplementary Table 10.** Distribution of very low-fair perceived relevance of topics and skills across age tertiles

| **Topic, n (%)** | **All (n=346)** | **23-30 years (n=130)** | **31-42 years (n=101)** | **43-72 years (n=115)** | **P value** |
| --- | --- | --- | --- | --- | --- |
| Depression | 138 (39.9%) | 57 (43.8%) | 36 (35.6%) | 45 (39.1%) | 0.44 |
| MCI and dementia: screening | 174 (50.3%) | 68 (52.3%) | 46 (45.5%) | 60 (52.2%) | 0.53 |
| Dementia: management of BPSD | 185 (53.5%) | 74 (56.9%) | 53 (52.5%) | 58 (50.4%) | 0.58 |
| Assessing cognitive status | 246 (71.1%) | 98 (75.4%) | 69 (68.3%) | 79 (68.7%) | 0.39 |
| Assessing functional capacity | 252 (72.8%) | 99 (76.2%) | 73 (72.3%) | 80 (69.6%) | 0.51 |
| Assessing capacity to consent | 214 (61.8%) | 89 (68.5%) | 62 (61.4%) | 63 (54.8%) | 0.09 |
| Advance healthcare directives | 252 (72.8%) | 105 (80.8%) | 74 (73.3%) | 73 (63.5%) | 0.01 |
| Palliative & End of Life care | 221 (63.9%) | 92 (70.8%) | 58 (57.4%) | 71 (61.7%) | 0.09 |
| Chronic pain | 136 (39.3%) | 56 (43.1%) | 34 (33.7%) | 46 (40%) | 0.34 |
| Geriatric rehabilitation | 267 (77.2%) | 108 (83.1%) | 77 (76.2%) | 82 (71.3%) | 0.09 |
| Healthy ageing | 135 (39%) | 54 (41.5%) | 35 (34.7%) | 46 (40%) | 0.55 |
| Orthogeriatrics | 251 (72.5%) | 101 (77.7%) | 71 (70.3%) | 79 (68.7%) | 0.24 |
| Oncogeriatrics | 208 (60.1%) | 88 (67.7%) | 57 (56.4%) | 63 (54.8%) | 0.08 |
| Gerodontology | 265 (76.6%) | 103 (79.2%) | 77 (76.2%) | 85 (73.9%) | 0.62 |
| Nutritional assessment & malnutrition | 207 (59.8%) | 85 (65.4%) | 53 (52.5%) | 69 (60%) | 0.14 |
| Dysphagia | 239 (69.1%) | 95 (73.1%) | 62 (61.4%) | 82 (71.3%) | 0.13 |
| Polypharmacy & deprescribing | 101 (29.2%) | 43 (33.1%) | 27 (26.7%) | 31 (27%) | 0.47 |
| Urinary & faecal incontinence | 210 (60.7%) | 84 (64.6%) | 57 (56.4%) | 69 (60%) | 0.44 |
| Hearing & vision impairments | 241 (69.7%) | 92 (70.8%) | 67 (66.3%) | 82 (71.3%) | 0.69 |
| Skin care & pressure ulcers | 173 (50%) | 69 (53.1%) | 49 (48.5%) | 55 (47.8%) | 0.67 |
| Ageism | 230 (66.5%) | 89 (68.5%) | 68 (67.3%) | 73 (63.5%) | 0.70 |
| Resilience & diversity | 160 (46.2%) | 65 (50%) | 41 (40.6%) | 54 (47%) | 0.36 |
| Abuse of older people | 264 (76.3%) | 105 (80.8%) | 75 (74.3%) | 84 (73%) | 0.31 |
| Assessing older people in ED | 301 (87%) | 115 (88.5%) | 86 (85.1%) | 100 (87%) | 0.76 |
| Communication: older people & caregivers | 203 (58.7%) | 81 (62.3%) | 56 (55.4%) | 66 (57.4%) | 0.54 |
| Communication: multidisciplinary team | 172 (49.7%) | 67 (51.5%) | 40 (39.6%) | 65 (56.5%) | 0.04 |
| Biology of ageing | 161 (46.5%) | 70 (53.8%) | 40 (39.6%) | 51 (44.3%) | 0.08 |
| Frailty | 177 (51.2%) | 73 (56.2%) | 49 (48.5%) | 55 (47.8%) | 0.35 |
| CGA | 204 (59%) | 86 (66.2%) | 60 (59.4%) | 58 (50.4%) | 0.04 |
| Falls & mobility | 167 (48.3%) | 71 (54.6%) | 42 (41.6%) | 54 (47%) | 0.14 |
| Bone health | 111 (32.1%) | 43 (33.1%) | 29 (28.7%) | 39 (33.9%) | 0.68 |
| Sarcopenia | 165 (47.7%) | 72 (55.4%) | 40 (39.6%) | 53 (46.1%) | 0.05 |
| Delirium | 185 (53.5%) | 70 (53.8%) | 49 (48.5%) | 66 (57.4%) | 0.42 |
| Depression | 138 (39.9%) | 57 (43.8%) | 36 (35.6%) | 45 (39.1%) | 0.44 |

Abbreviations: CGA: Comprehensive Geriatric Assessment; MCI: mild cognitive impairment; BPSD: behavioural and psychiatric symptoms of dementia; ED: Emergency Department.

**Supplementary Table 11.** Distribution of very low-fair perceived relevance of topics and skills in the overall population and among men and women.

| **Topic, n (%)** | **Overall (n=346)** | **Men (n=80)** | **Women (n=245)** | **P value** |
| --- | --- | --- | --- | --- |
| Depression | 138 (40%) | 25 (41.7%) | 113 (39.6%) | 0.88 |
| MCI and dementia: screening | 174 (50.4%) | 30 (50%) | 144 (50.5%) | 0.99 |
| Dementia: management of BPSD | 185 (53.6%) | 31 (51.7%) | 154 (54%) | 0.85 |
| Assessing cognitive status | 246 (71.3%) | 43 (71.7%) | 203 (71.2%) | 0.99 |
| Assessing functional capacity | 252 (73%) | 42 (70%) | 210 (73.7%) | 0.67 |
| Assessing capacity to consent | 214 (62%) | 35 (58.3%) | 179 (62.8%) | 0.62 |
| Advance healthcare directives | 252 (73%) | 38 (63.3%) | 214 (75.1%) | 0.09 |
| Palliative & End of Life care | 221 (64.1%) | 36 (60%) | 185 (64.9%) | 0.57 |
| Chronic pain | 136 (39.4%) | 25 (41.7%) | 111 (38.9%) | 0.81 |
| Geriatric rehabilitation | 266 (77.1%) | 42 (70%) | 224 (78.6%) | 0.20 |
| Healthy ageing | 135 (39.1%) | 28 (46.7%) | 107 (37.5%) | 0.24 |
| Orthogeriatrics | 251 (72.8%) | 40 (66.7%) | 211 (74%) | 0.31 |
| Oncogeriatrics | 208 (60.3%) | 35 (58.3%) | 173 (60.7%) | 0.84 |
| Gerodontology | 265 (76.8%) | 47 (78.3%) | 218 (76.5%) | 0.89 |
| Nutritional assessment & malnutrition | 207 (60%) | 34 (56.7%) | 173 (60.7%) | 0.66 |
| Dysphagia | 239 (69.3%) | 41 (68.3%) | 198 (69.5%) | 0.98 |
| Polypharmacy & deprescribing | 101 (29.3%) | 17 (28.3%) | 84 (29.5%) | 0.98 |
| Qn_2.26_Urinary_faecal_incontinence_Relevance | 210 (60.9%) | 37 (61.7%) | 173 (60.7%) | 0.99 |
| Hearing & vision impairments | 241 (69.9%) | 41 (68.3%) | 200 (70.2%) | 0.90 |
| Skin care & pressure ulcers | 173 (50.1%) | 34 (56.7%) | 139 (48.8%) | 0.33 |
| Ageism | 229 (66.4%) | 38 (63.3%) | 191 (67%) | 0.69 |
| Resilience & diversity | 160 (46.4%) | 29 (48.3%) | 131 (46%) | 0.85 |
| Abuse of older people | 263 (76.2%) | 44 (73.3%) | 219 (76.8%) | 0.68 |
| Assessing older people in ED | 301 (87.2%) | 55 (91.7%) | 246 (86.3%) | 0.36 |
| Communication: older people & caregivers | 203 (58.8%) | 37 (61.7%) | 166 (58.2%) | 0.73 |
| Communication: multidisciplinary team | 172 (49.9%) | 30 (50%) | 142 (49.8%) | 0.99 |
| Biology of ageing | 161 (46.7%) | 29 (48.3%) | 132 (46.3%) | 0.89 |
| Frailty | 177 (51.3%) | 32 (53.3%) | 145 (50.9%) | 0.84 |
| CGA | 204 (59.1%) | 36 (60%) | 168 (58.9%) | 0.99 |
| Falls & mobility | 167 (48.4%) | 26 (43.3%) | 141 (49.5%) | 0.47 |
| Bone health | 111 (32.2%) | 17 (28.3%) | 94 (33%) | 0.58 |
| Sarcopenia | 165 (47.8%) | 28 (46.7%) | 137 (48.1%) | 0.96 |
| Delirium | 185 (53.6%) | 31 (51.7%) | 154 (54%) | 0.85 |
| Depression | 138 (40%) | 25 (41.7%) | 113 (39.6%) | 0.88 |

Abbreviations: CGA: Comprehensive Geriatric Assessment; MCI: mild cognitive impairment; BPSD: behavioural and psychiatric symptoms of dementia; ED: Emergency Department.

**Supplementary Table 12.** Distribution of very low-fair perceived relevance of topics and skills across age tertiles

| **Topic, n (%)** | **All (n=346)** | **23-30 years (n=130)** | **31-42 years (n=101)** | **43-72 years (n=115)** | **P value** |
| --- | --- | --- | --- | --- | --- |
| Depression | 94 (27.2%) | 34 (26.2%) | 27 (26.7%) | 33 (28.7%) | 0.90 |
| MCI and dementia: screening | 117 (33.8%) | 44 (33.8%) | 32 (31.7%) | 41 (35.7%) | 0.83 |
| Dementia: management of BPSD | 126 (36.4%) | 47 (36.2%) | 37 (36.6%) | 42 (36.5%) | 1.00 |
| Assessing cognitive status | 171 (49.4%) | 66 (50.8%) | 49 (48.5%) | 56 (48.7%) | 0.93 |
| Assessing functional capacity | 182 (52.6%) | 70 (53.8%) | 56 (55.4%) | 56 (48.7%) | 0.57 |
| Assessing capacity to consent | 165 (47.7%) | 61 (46.9%) | 53 (52.5%) | 51 (44.3%) | 0.48 |
| Advance healthcare directives | 186 (53.8%) | 77 (59.2%) | 51 (50.5%) | 58 (50.4%) | 0.29 |
| Palliative & End of Life care | 145 (41.9%) | 59 (45.4%) | 40 (39.6%) | 46 (40%) | 0.60 |
| Chronic pain | 95 (27.5%) | 37 (28.5%) | 26 (25.7%) | 32 (27.8%) | 0.89 |
| Geriatric rehabilitation | 191 (55.2%) | 67 (51.5%) | 60 (59.4%) | 64 (55.7%) | 0.49 |
| Healthy ageing | 94 (27.2%) | 34 (26.2%) | 30 (29.7%) | 30 (26.1%) | 0.79 |
| Orthogeriatrics | 180 (52%) | 71 (54.6%) | 53 (52.5%) | 56 (48.7%) | 0.65 |
| Oncogeriatrics | 149 (43.1%) | 57 (43.8%) | 45 (44.6%) | 47 (40.9%) | 0.84 |
| Gerodontology | 210 (60.7%) | 78 (60%) | 66 (65.3%) | 66 (57.4%) | 0.48 |
| Nutritional assessment & malnutrition | 150 (43.4%) | 57 (43.8%) | 43 (42.6%) | 50 (43.5%) | 0.98 |
| Dysphagia | 177 (51.2%) | 68 (52.3%) | 47 (46.5%) | 62 (53.9%) | 0.53 |
| Polypharmacy & deprescribing | 72 (20.8%) | 30 (23.1%) | 15 (14.9%) | 27 (23.5%) | 0.21 |
| Urinary & faecal incontinence | 164 (47.4%) | 64 (49.2%) | 46 (45.5%) | 54 (47%) | 0.85 |
| Hearing & vision impairments | 176 (50.9%) | 64 (49.2%) | 50 (49.5%) | 62 (53.9%) | 0.73 |
| Skin care & pressure ulcers | 127 (36.7%) | 48 (36.9%) | 35 (34.7%) | 44 (38.3%) | 0.86 |
| Ageism | 179 (51.7%) | 63 (48.5%) | 59 (58.4%) | 57 (49.6%) | 0.28 |
| Resilience & diversity | 119 (34.4%) | 47 (36.2%) | 35 (34.7%) | 37 (32.2%) | 0.81 |
| Abuse of older people | 187 (54%) | 64 (49.2%) | 59 (58.4%) | 64 (55.7%) | 0.35 |
| Assessing older people in ED | 228 (65.9%) | 81 (62.3%) | 65 (64.4%) | 82 (71.3%) | 0.31 |
| Communication: older people & caregivers | 157 (45.4%) | 62 (47.7%) | 44 (43.6%) | 51 (44.3%) | 0.79 |
| Communication: multidisciplinary team | 130 (37.6%) | 50 (38.5%) | 29 (28.7%) | 51 (44.3%) | 0.06 |
| Biology of ageing | 107 (30.9%) | 37 (28.5%) | 34 (33.7%) | 36 (31.3%) | 0.69 |
| Frailty | 127 (36.7%) | 46 (35.4%) | 40 (39.6%) | 41 (35.7%) | 0.77 |
| CGA | 145 (41.9%) | 57 (43.8%) | 45 (44.6%) | 43 (37.4%) | 0.48 |
| Falls & mobility | 125 (36.1%) | 46 (35.4%) | 37 (36.6%) | 42 (36.5%) | 0.98 |
| Bone health | 90 (26%) | 28 (21.5%) | 26 (25.7%) | 36 (31.3%) | 0.22 |
| Sarcopenia | 121 (35%) | 48 (36.9%) | 32 (31.7%) | 41 (35.7%) | 0.70 |
| Delirium | 120 (34.7%) | 42 (32.3%) | 32 (31.7%) | 46 (40%) | 0.34 |

Abbreviations: CGA: Comprehensive Geriatric Assessment; MCI: mild cognitive impairment; BPSD: behavioural and psychiatric symptoms of dementia; ED: Emergency Department.

**Supplementary Table 13.** Distribution of very low-fair perceived relevance of topics and skills among men and women

| **Topic, n (%)** | **Overall (n=346)** | **Men (n=80)** | **Women (n=245)** | **P value** |
| --- | --- | --- | --- | --- |
| Depression | 94 (27.2%) | 21 (35%) | 73 (25.6%) | 0.19 |
| MCI and dementia: screening | 117 (33.9%) | 25 (41.7%) | 92 (32.3%) | 0.21 |
| Dementia: management of BPSD | 126 (36.5%) | 28 (46.7%) | 98 (34.4%) | 0.10 |
| Assessing cognitive status | 171 (49.6%) | 34 (56.7%) | 137 (48.1%) | 0.29 |
| Assessing functional capacity | 182 (52.8%) | 34 (56.7%) | 148 (51.9%) | 0.60 |
| Assessing capacity to consent | 165 (47.8%) | 29 (48.3%) | 136 (47.7%) | 10.99 |
| Advance healthcare directives | 186 (53.9%) | 31 (51.7%) | 155 (54.4%) | 0.81 |
| Palliative & End of Life care | 145 (42%) | 23 (38.3%) | 122 (42.8%) | 0.62 |
| Chronic pain | 95 (27.5%) | 21 (35%) | 74 (26%) | 0.21 |
| Geriatric rehabilitation | 191 (55.4%) | 32 (53.3%) | 159 (55.8%) | 0.84 |
| Healthy ageing | 94 (27.2%) | 19 (31.7%) | 75 (26.3%) | 0.49 |
| Orthogeriatrics | 180 (52.2%) | 29 (48.3%) | 151 (53%) | 0.61 |
| Oncogeriatrics | 149 (43.2%) | 26 (43.3%) | 123 (43.2%) | 0.99 |
| Gerodontology | 210 (60.9%) | 35 (58.3%) | 175 (61.4%) | 0.77 |
| Nutritional assessment & malnutrition | 150 (43.5%) | 26 (43.3%) | 124 (43.5%) | 0.99 |
| Dysphagia | 177 (51.3%) | 34 (56.7%) | 143 (50.2%) | 0.44 |
| Polypharmacy & deprescribing | 72 (20.9%) | 13 (21.7%) | 59 (20.7%) | 0.99 |
| Urinary & faecal incontinence | 164 (47.5%) | 27 (45%) | 137 (48.1%) | 0.77 |
| Hearing & vision impairments | 176 (51%) | 33 (55%) | 143 (50.2%) | 0.59 |
| Skin care & pressure ulcers | 127 (36.8%) | 26 (43.3%) | 101 (35.4%) | 0.31 |
| Ageism | 178 (51.6%) | 33 (55%) | 145 (50.9%) | 0.66 |
| Resilience & diversity | 119 (34.5%) | 25 (41.7%) | 94 (33%) | 0.26 |
| Abuse of older people | 187 (54.2%) | 35 (58.3%) | 152 (53.3%) | 0.57 |
| Assessing older people in ED | 228 (66.1%) | 41 (68.3%) | 187 (65.6%) | 0.80 |
| Communication: older people & caregivers | 157 (45.5%) | 27 (45%) | 130 (45.6%) | 0.99 |
| Communication: multidisciplinary team | 130 (37.7%) | 26 (43.3%) | 104 (36.5%) | 0.40 |
| Biology of ageing | 107 (31%) | 19 (31.7%) | 88 (30.9%) | 0.99 |
| Frailty | 127 (36.8%) | 24 (40%) | 103 (36.1%) | 0.68 |
| CGA | 145 (42%) | 27 (45%) | 118 (41.4%) | 0.71 |
| Falls & mobility | 125 (36.2%) | 23 (38.3%) | 102 (35.8%) | 0.82 |
| Bone health | 90 (26.1%) | 17 (28.3%) | 73 (25.6%) | 0.78 |
| Sarcopenia | 121 (35.1%) | 27 (45%) | 94 (33%) | 0.10 |
| Delirium | 120 (34.8%) | 23 (38.3%) | 97 (34%) | 0.63 |

Abbreviations: CGA: Comprehensive Geriatric Assessment; MCI: mild cognitive impairment; BPSD: behavioural and psychiatric symptoms of dementia; ED: Emergency Department.
